# Orismilast, a Potent and Selective PDE4B/D Inhibitor, Reduces Protein Levels of Key Disease Driving Cytokines in the Skin of Patients With Plaque Psoriasis

**DOI:** 10.1111/exd.70153

**Published:** 2025-09-19

**Authors:** Richard B. Warren, Anne Weiss, Jakob Felding, Morten O. A. Sommer, Sandra Garcet, James G. Krueger

**Affiliations:** ^1^ Dermatology Centre, Salford Royal NHS Foundation Trust, Manchester NIHR Biomedical Research Centre The University of Manchester Manchester UK; ^2^ UNION Therapeutics A/S Hellerup Denmark; ^3^ Novo Nordisk Foundation Center for Biosustainability Technical University of Denmark (DTU) Lyngby Denmark; ^4^ Laboratory of Investigative Dermatology The Rockefeller University New York New York USA

**Keywords:** biomarker, IASOS, orismilast, PDE4, PDE4 B/D selective, psoriasis

## Abstract

Minimally invasive sampling of the skin using tape strips for conducting biomarker research is a growing research area in medical dermatology. The goal of this study was to utilise tape strip sampling to investigate changes in protein skin levels of psoriasis patients after oral treatment with orismilast (a PDE4B/D inhibitor). The proteins were measured in extracts of tape‐strip samples taken from the skin of patients with moderate–severe psoriasis participating in a 16‐week Ph2b study (IASOS). The proteins were measured using the Olink technology or an ELISA assay. Our results show that protein levels of multiple proteins (32/71) were upregulated at baseline in the lesional skin compared to non‐lesional skin, including three key biomarkers of the psoriasis disease pathology (IL‐17A, CCL20 and TNFα). The protein levels of these three biomarkers were significantly reduced at Week 16, reaching a percent reduction of 52% and 51% for IL‐17A, 66% and 60% for TNFα, and 41% and 54% for CCL20 for the two doses analysed (20 and 30 mg bid, respectively). In addition, we observed that the clinical response of a 75% reduction in PASI (PASI75) was associated with a 98% reduction in IL‐17A protein levels in lesional skin, irrespective of the orismilast dose. In summary, a significant reduction of key proteins related to the T_H_17 axis and T_H_1 axis was observed in the skin of psoriasis patients after treatment with oral orismilast, supporting the observed clinical effect. Finally, this constitutes the first report where protein levels from the skin of psoriasis patients are quantified using tape strips as a minimally invasive skin sampling technology in combination with the Olink technology.

**Trial Registration:** ClinicalTrials.gov identifier: NCT05190419

## 1 Introduction

Plaque psoriasis (psoriasis) is a chronic autoimmune disease that affects approximately 90% of psoriasis patients and nearly 125 million people globally [1]. The clinical manifestation of psoriasis is characterised by well‐defined, red, elevated skin plaques covered with silvery scales. Although psoriasis is visible on the skin, it is driven by systemic inflammation and is associated with other inflammatory conditions and comorbidities, including cardiovascular diseases, psoriatic arthritis, inflammatory bowel disease, depression and obesity [2]. In addition, accumulating evidence suggests an increased mortality rate among patients diagnosed with psoriasis [3].

There is no cure for psoriasis, and multiple factors are important to consider when prescribing a drug for treating psoriasis, including disease severity, comorbidities, safety and cost. Topical therapy is not well‐suited for patients with moderate–severe psoriasis, as large body surface areas are affected. The discovery of the interleukin (IL)‐23/T_H_17 pathway as a central disease‐driving pathway has facilitated the development of highly efficacious injectable antibodies targeting cytokines and cytokine receptors of the IL‐23/T_H_17 immune axis (e.g., anti‐IL‐17A, anti‐IL17R‐ and anti‐IL23 antibodies), but primary and secondary treatment failures or inadequate responses to initial treatment and fear of needles can be a challenge [4, 5]. Oral therapy is still preferred by many patients and prescribers, but the orally available immunosuppressive drugs (e.g., methotrexate, ciclosporin, JAK1—and TYK2 inhibitors) involve safety monitoring [6]. The phosphodiesterase 4 (PDE4) inhibitor apremilast represents a safe, oral drug; however, the level of efficacy in psoriasis is limited [7, 8]. A high unmet medical need for safe and more effective orally available drugs still exists [9]. Orismilast is a potent PDE4 inhibitor with high selectivity for the PDE4B and PDE4D subtypes and demonstrated significant efficacy versus placebo at Week 16 in patients with moderate‐to‐severe psoriasis, both for percentage change in PASI and proportions of patients achieving PASI75 and PASI90 [10, 11, 12]. Based on these data, orismilast has the potential to be a safe oral drug that provides greater efficacy than less potent pan‐PDE4 inhibitors such as apremilast.

In this article, we report skin biomarker data based on tape strip samples from patients with psoriasis who were participating in the Phase 2b IASOS trial [12]. The main objective of the biomarker study was to investigate the effect of orismilast on a broad spectrum of inflammatory markers in psoriatic skin lesions and constitutes the first report where protein levels from the skin of psoriasis patients are quantified using tape strips as a minimally invasive skin sampling technology in combination with the Olink technology.

## 2 Methods

### 2.1 Clinical Trial Outline

Skin tape strip samples were taken from lesional and non‐lesional skin at baseline and lesional skin at Week 16 of patients participating in a multicenter, randomised, double‐blinded, placebo‐controlled, phase 2b, dose‐ranging study assessing oral orismilast in adults with moderate‐to‐severe plaque psoriasis (IASOS study) [12]. This study was conducted in accordance with the International Council for Harmonisation of Technical Requirements for Pharmaceuticals for Human Use, Declaration of Helsinki and with the approval of National Independent Ethics Committees.

### 2.2 Sampling and Methods

**FIGURE 1:**
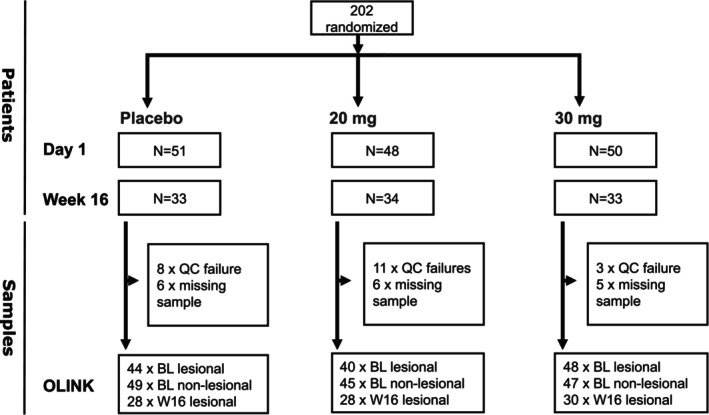
Sample flow of tape strips for OLINK analysis. The tape‐strip sampling was part of the Phase 2b trial (IASOS). Week 16 samples were only analysed for treatment completers. The numbers in the top part of the figure represent the number of patients that were eligible for sample collection, whereas the numbers in the bottom part reflect the numbers of samples that were missing (due to QC failure or missed sampling) and finally the numbers of samples used in analysis. BL, Baseline; QC, Quality control; W16, Week 16.

Approximately 20 consecutive stratum corneum samples were collected from the same skin site by tape stripping (D‐squames; Monaderm DS100) of both lesional and non‐lesional skin at baseline and lesional skin at Week 16. To extract the proteins from the tapes, the first 10 tapes were pooled together and used for subsequent protein extraction by incubation in 800 μL of PBS containing 0.2% Triton X‐100 together with a mixture of protease inhibitors and incubated overnight at 4°C under stirring at 1400 rpm in a thermo mixer (Eppendorf). The insoluble material was removed by filtration on 0.22 μm filters by centrifugation. The protein extracts were analysed using the Olink technology (Target 96 Inflammation panel) and an IL‐23 ELISA assay (V‐Plex MSD). Protein extracts from patients treated with placebo, 20 mg orismilast bid, and 30 mg orismilast bid were analysed. Samples from the 40 mg bid arm were excluded from the OLINK and ELISA analyses due to tolerability issues and the relatively high drop‐out rate observed in this arm hindering 40 mg bid dosing from being used in future clinical development in psoriasis [12]. The sample flow of tape strip samples for the OLINK analysis is displayed in Figure 1 and for IL‐23 in Figure S1.

The biomarker population is a representative subset of the ITT population of the IASOS trial as the population size and the PASI score at baseline are comparable to the ITT population, across treatment arms (Table S1) [12].

### 2.3 Statistical Analysis

Proteomic profile was estimated under R limma package framework using mixed‐effect linear models to estimate and compare the least squares means of the different groups and to calculate comparison's fold changes as well. Treatment (Placebo, Orismilast 20 mg, Orismilast 30 mg), tissue (nonlesional/lesional skin), time point (baseline/Week 16) and clinical response (PASI75 responder, non‐responder) were considered fixed factors while random effect related to each subject was included. We employed mixed‐effects models to account for non‐independence in the data due to repeated measures (e.g., timepoints and tissue types) and hierarchical structures (e.g., responders vs. non‐responders within treatment groups). These models incorporate both fixed and random effects, enhancing robustness and accommodating missing data. Additionally, they allow for the estimation of individual‐ or group‐level variation, providing insights beyond overall averages.

Tape strip protein levels were log_2_‐transformed and a mixed‐effect linear model was fitted to the data. A Student *t*‐test was performed for statistical comparisons and *p*‐values were corrected for multiple comparisons (false discovery rate [FDR]) using the Benjamini & Hochberg (1995) method. Proteins with an adjusted *p*‐value (FDR) of < 0.05 and a fold change absolute value > 1.2 were defined as differentially expressed, as used in a similar study by Navrazhina et al. [13] The disease proteome was defined at baseline (lesional vs. non‐lesional) as well as treatment effect (lesional post treatment vs. baseline). Proteins with over 90% of measurements below the limit of detection were excluded from the analysis (see Table S2 for list of proteins). IL23 data were log_2_‐transformed for statistical analysis and analysed identically to the OLINK data.

Proteins in the 20 and 30 mg bid orismilast groups as well as the placebo group were additionally analysed in a stratified manner by PASI75 response (clinical response of a 75% reduction in PASI at Week 16) using *t*‐test statistical testing. Selected markers were similarly analysed in PASI90 responders versus non‐responders following orismilast treatment using *t*‐test statistical testing with a *p*‐value of < 0.05 considered statistically significant.

Outcomes (estimates and standard error of mean) of conducted statistical tests are reported in Tables S3 and S4.

## 3 Results

The reported skin biomarker data are from psoriasis patients enrolled in a Ph2b dose‐ranging study (IASOS). In this study, orismilast showed significant improvements in the primary end point, percentage change in Psoriasis Area and Severity Index (PASI), from baseline to Week 16 (orismilast −52.6% (20 mg bid) to −61.2% (30 mg bid) and placebo, −17.3%; all *p* < 0.001). Furthermore, a greater proportion of patients receiving orismilast achieved PASI75 (39.5% and 49.0%; *p* < 0.05) and PASI90 (24.1% and 22.0%; *p* < 0.05 for 20 mg) versus placebo (PASI75, 16.5% and PASI90, 8.3%) at Week 16 [12].

Tape strip samples of lesional and non‐lesional skin were processed and analysed with the OLINK proteomic assay before and after treatment with oral orismilast, measuring 70 proteins in the OLINK Inflammation panel and quantifying IL‐23 levels with MSD. Changes in biomarker levels were assessed using the criteria of a fold change > |1.2| and FDR < 0.05. Orismilast therapy induced an overall change of 29% (16/56 biomarkers) and 46% (26/56 biomarkers) in lesional proteins at Week 16 for 20 and 30 mg bid, respectively. In the placebo arm, we found that only 4% (2/56 biomarkers) were differentially expressed at Week 16 (Figure S2). In addition, we identified 32 up‐regulated lesional proteins at baseline, of which 28% (9/32, 20 mg bid) and 47% (15/32, 30 mg bid) were significantly reduced following treatment with orismilast.

**FIGURE 2:**
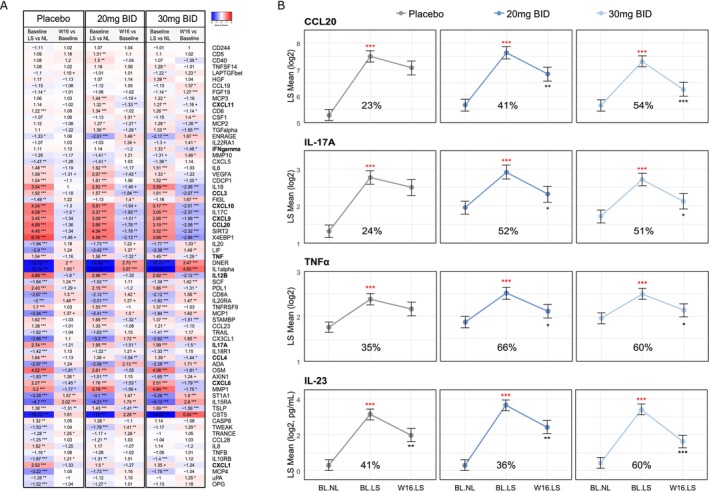
Effect on skin proteins in psoriasis patients following 16 weeks of treatment with placebo or orismilast. (A) Heatmap displays fold changes of skin proteins following placebo, 20 and 30 mg bid, and comparing lesional (LS) and non‐lesional skin (NL) at baseline and lesional skin at Week 16 (W16) versus lesional skin (LS) at baseline. Bolded proteins relate to T_H_17 cells and T_H_1 cells. Raw data can be found in Table S3. (B) Least square means of key disease markers at baseline and following 16 weeks of treatment. Red asterisks denote statistical comparison of baseline LS versus baseline NL levels and black asterisks denote statistical comparison of lesional skin levels at Week 16 versus baseline LS. Statistical comparisons were conducted comparing change in protein levels of Week 16 LS versus baseline LS between these groups. The respective fold changes can be found in panel A (for CCL20, IL‐17A, TNFα). Percent improvement was calculated per group and protein: Abs (Week 16 lesional − baseline lesional)/abs (baseline lesional − baseline non lesional). ***p < 0.001, **p < 0.01, *p < 0.05. bid, twice‐daily; BL, Baseline.

Proteins related to the T_H_1 and T_H_17 immune axis were significantly upregulated in lesional skin versus non‐lesional skin at baseline, whereas key cytokines related to T_H_2 cells (e.g., IL‐4 and IL‐13) could not be detected in this patient population (Figure 2A). At Week 16, an immunomodulatory effect of orismilast across several immune axes was observed as demonstrated by a significant reduction in lesional protein levels related to T_H_17 (e.g., IL‐23, IL‐17A, CCL20 and IL‐12B), T_H_1 (e.g., TNFα, IFNγ, CXCL9 and CXCL10) and epithelial inflammation (e.g., IL‐17C) (Table 1). In contrast, most of the proteins were not significantly modulated in samples from placebo patients. Furthermore, key markers of the psoriasis disease pathology were significantly reduced in the two active arms (20 mg/30 mg) reaching a percent improvement of 52% versus 51% (IL‐17A), 41% versus 54% (CCL20) and 66% versus 60% (TNFα) at Week 16, as seen in Figure 2B. The improvement in these markers was substantially higher in the two active arms compared to placebo; however, the difference did not reach statistical significance.

**TABLE 1:** Effect on significantly up‐regulated proteins at baseline after 16 weeks treatment with orismilast.

Pathway	Protein	20 mg bid	30 mg bid
		Week 16 lesional versus baseline lesional	
Innate Immunity	IL‐18	−1.46	−2.06***
Epithelial inflammation	IL‐17C	−1.93**	−2.37***
MMP	MMP1	−1.59	−1.75*
T_H_1‐related	TNFα	−1.32*	−1.29*
	CCL3	−1.84***	−2.07***
	CCL4	−1.54**	−1.44*
	CXCL6	−1.53*	−1.79***
	CXCL9	−1.51*	−1.99***
	CXCL10	−1.54	−2.01***
	CXCL11	−1.33**	−1.18
T_H_17‐related	IL‐12B	−1.32	−2.12***
	IL‐17A	−1.51*	−1.50*
	CCL20	−1.76**	−2.08***
	TNFα	−1.32*	−1.29*
	IL23	−2.3**	−3.4***
	IFNγ	−1.2	−1.48*

*Note:* Fold changes of proteins comparing lesional skin at Week 16 versus baseline. Proteins are sorted by pathway.

Abbreviations: bid, twice‐daily; LS, lesional; MMP, matrix‐metalloprotease.

* *p* < 0.05.

** *p* < 0.01.

*** *p* < 0.001.

**FIGURE 3:**
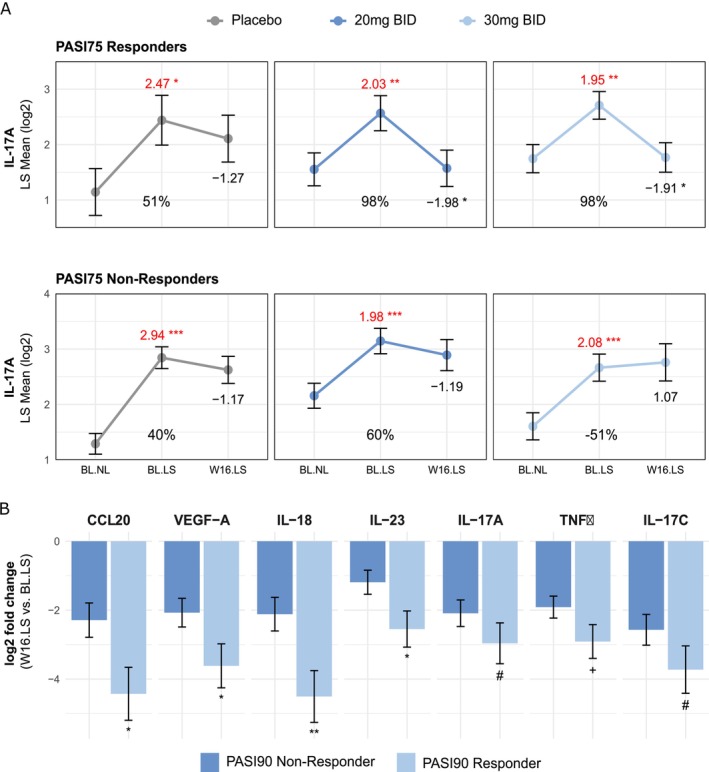
Biomarker changes stratified by clinical response. (A) IL‐17A levels stratified by PASI75 response for each treatment arm. Least square means at baseline and following 16 weeks of treatment are displayed. Fold changes and *p*‐value of (i) lesional (LS) versus non‐lesional (NL) skin levels at baseline (red) and (ii) lesional skin levels at Week 16 versus baseline (black) are included. Percent improvement, which is included in each panel, was calculated per group and protein: Abs (Week 16 lesional − baseline lesional)/abs (baseline lesional − baseline non lesional). Numbers of responders/non‐responders: Placebo = 8/25, 20 mg BID = 17/18, 30 mg BID = 13/22. (B) Log_2_ fold‐change with standard error of selected markers in PASI90 responders (*N* = 21) versus non‐responders (*N* = 49) following orismilast treatment. Statistical comparison of responders versus non‐responders using *t*‐test. ***p < 0.001, **p < 0.01. *p < 0.05, +*p* < 0.1, #*p* = 0.2. bid, twice‐daily; BL = Baseline; LS, lesional; NL, non‐lesional; PASI, Psoriasis Area and Severity Index; W16, Week 16. See Tables S4 and S5 for raw data.

In a subgroup analysis, we stratified patients in each treatment arm by PASI75 response and found 15 (20 mg bid) and 17 (30 mg bid) differentially expressed proteins (DEPs) that were downregulated at Week 16 in patients treated with orismilast. In contrast, no DEPs were identified in patients who were PASI75 non‐responders after treatment with orismilast or achieved PASI75 response in the placebo arm (Table S6). IL‐17A has been identified as one of the key disease drivers of psoriasis [14]. In our study, we found a significant reduction of lesional IL‐17A in the PASI75 responders of the orismilast treatment groups, but not in the PASI75 non‐responders (Figure 3A). The degree of IL‐17A reduction was similar in responders irrespective of the orismilast dose, with lesional levels at Week 16 being comparable to non‐lesional levels and showing a 98% improvement. Furthermore, additional proteins related to the T_H_1 and T_H_17 immune axis followed a similar trend (Figure S3). Given the similar effect level of the two orismilast doses, we combined data of the 20 and 30 mg arms to enrich the dataset for an additional subgroup analysis. Stratifying patients by PASI90 response, we found that IL‐23, CCL20, IL‐18 and VEGF‐A were significantly reduced (*p* < 0.05) in PASI90 responders versus non‐responders. IL‐17A, TNFα and IL‐17C were also reduced but less strongly (Figure 3B).

## 4 Discussion

A primary objective of the biomarker part of the clinical Ph2b study was to compare differentially expressed inflammatory proteins in the skin of psoriasis patients at baseline to treatment with oral orismilast for 16 weeks using clinically relevant doses (20 and 30 mg bid).

We found a significant reduction in lesional levels of the key psoriasis disease drivers, IL‐23 and IL‐17A, at Week 16 following 20 and 30 mg orismilast, which supports the clinical effect observed in the Phase 2b study [12]. Furthermore, PASI75 responders of the orismilast treated groups showed a markedly different reduction in IL‐17A protein levels compared to placebo. In fact, a clinical response of a 75% reduction in PASI was associated with a 98% improvement in IL‐17A levels, irrespective of the orismilast dose. The limited additional benefit of 30 mg bid compared to 20 mg bid is in alignment with the observed clinical data, where the two doses differ in absolute PASI and PASI75 response only numerically [12].

IL‐17A levels showed substantially greater improvement in PASI75 responders compared to non‐responders; however, even among non‐responders, reductions of up to 60% in IL‐17A were observed across treatment arms, which seem to be in alignment with data from a Tyk2 inhibitor where a reduction of > 80% in IL‐17A/F or KRT16 was required to reach a meaningful histological and clinical response, respectively [15]. In addition, considerable variability in biomarker changes among non‐responders, along with the absence of differentially expressed proteins at Week 16, was observed.

Beyond IL‐17A and IL‐23, we observed a broad immunomodulatory effect of orismilast across the T_H_1 (e.g., TNFα, CXCL10, CXCL9) and T_H_17 (e.g., IL‐17A, IL‐23, IL‐12B, CCL20) immune axes. This is in agreement with several previous biomarker studies of the PDE4 inhibitor apremilast in psoriasis patients, showing a significant modulation of T_H_1 and T_H_17 cytokines involved in the pathogenesis of psoriasis (e.g., IL‐17A/F, IL‐22 and TNFα) [16, 17, 18]. Consistently across the apremilast studies, the most pronounced inhibition was, however, observed for IL‐17A and IL‐17F, which further showed the strongest correlations with PASI improvements following treatment with apremilast [16, 17, 18]. Predictive modelling found IL‐17 as the most important predictor of PASI improvement to apremilast, and demonstrated synergistic effects of IL‐17, IL‐22 and TNFα on PASI response [19]. Thus, the inhibition of the IL‐17 axis is thought to be an important mechanism through which PDE4 inhibitors exert their anti‐inflammatory effects in patients with psoriasis. Despite these mechanistic data published from studies with apremilast, a need for comprehensive profiling of the broad effect of PDE4 inhibition in psoriasis remains, especially regarding proteomic changes in the skin.

In agreement with studies of apremilast, we observed a greater reduction of lesional IL‐17A than TNFα following orismilast at Week 16, suggesting an effect beyond the classical TNFα pathway and linked to the T_H_17 pathway. This is further supported by a strong and significant reduction of cytokines related to IL‐17 inhibition (e.g., IL‐23, CCL20, IL‐12B), but only a weak reduction of cytokines related to the TNFα pathway, such as IL‐6 and IL‐8. In addition, we observed a significant correlation of lesional IL‐17A and IL‐17C levels with PASI improvement at Week 16, whereas Week 16 TNFα levels did not significantly correlate with PASI improvement. Finally, a significant reduction of lesional CCL20 and IL‐17C levels at Week 16 indicates an upstream blockade of IL‐17A and the effect likely occurring in keratinocytes. Thus, we hypothesise that TNFα positively contributes to the suppression of IL‐17A, potentially in synergy with IL‐17C, which is known to amplify inflammation in keratinocytes [20]. Given that PDE4 inhibitors were clinically efficacious in psoriasis and psoriatic arthritis patients (IL‐17/IL‐23 axis dominant diseases) [20, 21], but showed less effect in rheumatic arthritis patients [22] (TNFα axis dominant disease) further supports the hypothesis that PDE4 inhibition has a major impact on the pathogenic T_H_17 cell pathway. In addition, the significant reduction of IFNγ, CXCL10 and CXCL9 in PASI75 responders and significant correlation of all markers at Week 16 with PASI improvement indicates a direct modulation of cytokines related to the T_H_1 pathway or pathogenic T_H_17 cells co‐expressing IFNγ. Overall, the data presented in this study confirm the mechanistic understanding of the anti‐inflammatory effect seen with PDE4 inhibition. The broad anti‐inflammatory effect seen in psoriasis patients after treatment with orismilast is in alignment with preclinical data of orismilast in vitro and in vivo [10] and supports the mechanistic hypothesis of PDE4 inhibition impacting T cell receptor activation through the cAMP signalling pathway and keratinocyte activation, as reviewed by Pincelli et al. [17].

In our study, we observed a 20%–40% reduction in several key inflammatory markers among patients receiving placebo at Week 16; although no differentially expressed proteins were identified. The study design may have contributed to an elevated placebo effect: biomarker assessments were limited to study completers, potentially enriching the placebo group for patients who experienced some clinical improvement. While only non‐medicated emollients and low‐potency corticosteroids (restricted to the face, axillae and groin) were permitted, their impact on overall outcomes is likely minimal.

We observed a significant reduction in key inflammatory markers using orismilast and, most importantly, mirrored the transcriptomic and systemic biomarker changes seen with apremilast in psoriasis patients. Thus, tape stripping combined with OLINK can serve as a powerful tool to measure disease characteristics and the impact of a treatment modality on a patient's skin inflammatory signature. Employing tape stripping in a clinical trial setting enables the minimally invasive collection of skin samples in studies with a larger sample size (e.g., Phase 2b, Phase 3) and inclusion of a placebo group. In addition, the OLINK technology further allows for an ultra‐sensitive detection of a variety of proteins, which seems beneficial in the case of rather low levels of disease‐driving cytokines. However, while tape stripping offers a non‐invasive approach to quantify inflammatory proteins, it may not adequately capture inflammation within the dermis. An indirect comparison of our baseline data with the proteomic data from the study by Navrazhina et al. [13]—which analysed biopsies from healthy controls as well as lesional and non‐lesional skin of psoriasis patients using the same OLINK Inflammation Panel—shows that fewer analytes were quantifiable using tape stripping (70 out of 92) compared to biopsies (92 out of 92). Still, both methodologies similarly captured the dysregulation of key inflammatory markers of psoriasis in lesional skin. Proteins related to the central disease‐driving pathways of psoriasis, namely the T_H_1 (CCL3, CCL4, CXCL9, CXCL10, CXCL11, TNFα) and T_H_17 pathway (CXCL1, CCL20, IL‐17A, IL‐12B) were found elevated in lesional skin across both studies. Given the differences in study design and statistical approaches between the two studies, a more comprehensive comparison remains challenging. Therefore, a dedicated study would be necessary to fully elucidate the distinct proteomic profiles captured by tape stripping versus biopsy in psoriasis patients, as reported by Del Duca et al. [23] in patients with atopic dermatitis. To the best of our knowledge, this is the first study in psoriasis patients where lesional skin protein levels are quantified using tape strip sampling in combination with the OLINK technology. Tape stripping and skin proteomics have previously been employed to measure disease characteristics in psoriasis and atopic dermatitis patients [13, 24, 25]. However, in most studies, a therapeutic treatment was not included, and the two technologies were only combined in samples from patients with atopic dermatitis [26].

This study had limitations. The conclusions are based on data from a relatively small number of patients; however, the sample size is comparable and even slightly bigger than other biomarker studies. Only baseline and Week 16 samples were collected as the biomarker study was part of a dose‐range finding phase 2b study and not a dedicated biomarker study, where longitudinal sampling with more time points is sometimes collected. We have not generated gene expression data but focused on protein levels in the skin. Finally, a dedicated biomarker study in a larger cohort with longitudinal sampling would be needed to robustly assess predictors of response, resistance mechanisms and inter/intra‐patient variability, ultimately aiding in fully elucidating the mechanism of orismilast in psoriasis.

## 5 Conclusion

In conclusion, a significant reduction of key proteins related to the T_H_17 axis, T_H_1 axis and epithelial inflammation was observed in the skin of psoriasis patients after treatment with oral orismilast. The biomarker data obtained confirm the broad immunomodulatory effect observed in preclinical studies with orismilast and show a larger differentiation of orismilast to placebo than seen with clinical endpoints in psoriasis patients [10, 12]. Importantly, the study underlines tape strip sampling of the skin as a powerful and minimally invasive technology to obtain data on protein changes in clinical studies conducted in psoriasis patients.

## Author Contributions

R.B.W. contributed to conceptualisation, investigation, methodology, reviewing and editing. A.W. contributed to conceptualisation, data curation, formal analysis, investigation, methodology, project administration, visualisation, writing and reviewing and editing. J.F. contributed to conceptualisation, data curation, formal analysis, investigation, methodology, project administration, visualisation, writing and reviewing and editing. M.O.A.S. contributed to conceptualisation, investigation, methodology, reviewing and editing. S.G. contributed to conceptualisation, data curation, formal analysis, investigation, methodology, project administration, resources, software, supervision, visualisation, reviewing and editing the manuscript. J.G.K. contributed to conceptualisation, data curation, investigation, methodology, resources, supervision and reviewing and editing. All authors have read and approved the final manuscript.

## Conflicts of Interest

Dr. Richard B. Warren has received research grants from AbbVie, Almirall, Amgen, Celgene, Janssen, Lilly, LEO Pharma, Novartis, Pfizer, and UCB, and consulting fees from AbbVie, Almirall, Amgen, Arena, Astellas, Avillion, Biogen, Boehringer Ingelheim, Bristol Myers Squibb, Celgene, DiCE, GlaxoSmithKline, Janssen, Lilly, LEO Pharma, Novartis, Pfizer, Sanofi, Sun Pharma, UCB, and UNION therapeutics. Dr. Richard B. Warren was the lead investigator of the IASOS Phase 2b study. Dr. James G. Krueger has received grants paid to the institution from Novartis, Pfizer, Amgen, Lilly, Boehringer, Innovaderm, BMS, Janssen, Abbvie, Paraxel, LEO Pharma, Vitae, Akros, Regeneron, Allergan, Novan, Biogen MA, Sienna, UCB, Celgene, Botanix, Incyte, Avillion, Exicure; has received personal fees from Novartis, Pfizer, Amgen, Lilly, Boehringer, Biogen Idec, Abbvie, LEO Pharma, Escalier, Valeant, Aurigne, Allergan, Asana, UCB, Sienna, Celgene, Nimbus, Menlo, Aristea, Sanofi, Sun Pharma, Almirall, Arena, BMS, Ventyx, Aclaris, Galapagos, UNION therapeutics. A.W., J.F. and M.O.A.S. are all full‐time employees of UNION therapeutics. The study was funded by UNION therapeutics and orismilast is in clinical development by UNION therapeutics. Sandra Garcet declares no conflicts of interest.

## Supporting information


**Appendix S1:** exd70153‐sup‐0001‐AppendixS1.docx.
**Table S1:** Baseline characteristics of biomarker population as part of the Phase2b IASOS trial.
**Table S2:** List of proteins excluded from statistical analysis of OLINK data. Proteins with over 90% of measurements below limit of detection were excluded from the analysis.
**Table S3:** Outcomes of statistical tests conducted for full cohort.
Data for heatmap in Figure 2A. BL, Baseline; FCH, foldchange; LS, Lesional; NL, Non‐lesional; SEM, standard error of mean; W16, Week 16.
**Table S4:** Outcomes of statistical tests stratified by PASI75 response. Data for heatmap in Figure S3. BL, Baseline; FCH, foldchange; LS, Lesional; NL, Non‐lesional; SEM, standard error of mean; W16, Week 16.
**Table S5:** Outcomes of statistical tests stratified by PASI90 response. Data for Figure 3B. FCH, foldchange; SE, standard error.
**Table S6:** Number of differential expressed proteins in PASI75 Responders and Non‐responders. Differential expressed proteins were defined as |FCH| > 1.2 and false‐discovery rate < 0.05 for OLINK proteins. For IL23, a *p* value < 0.05 was used as a cutoff criterion, given that IL23 was quantified with a different methodology as it is not part of the OLINK 96 Inflammation panel.
**Figure S1:** Sample flow of tape strips for IL23 analysis. Week 16 samples were only analysed for treatment completers. BL, Baseline; BLOD, below limit of detection; W16, Week 16.
**Figure S2:** Differential expressed proteins before and after treatment with orismilast and placebo. Differential expressed proteins were defined as |FCH| > 1.2 and false‐discovery rate < 0.05 for OLINK proteins. For IL23, a *p* value < 0.05 was used as a cutoff criterion, given that IL23 was quantified with a different methodology as it is not part of the OLINK Inflammation panel.
**Figure S3:** Heatmap of biomarker response in PASI75 Responders and Non‐Responders. Heatmap is displaying fold changes of proteins which are elevated at baseline. BL, Baseline; NL, Nonlesional; LS, lesional; W16, Week 16.

## Data Availability

The data that supports the findings of this study are available in the Supporting Information of this article.
