# Supplementary material for: Orismilast, a Potent and Selective PDE4B/D Inhibitor, Reduces Protein Levels of Key Disease Driving Cytokines in the Skin of Patients With Plaque Psoriasis
Source: Exp Dermatol. 2025 Sep 19;34(9):e70153. doi: 10.1111/exd.70153 (PMC12447663; doi:10.1111/exd.70153)
Supplement: Supplementary file 1 — Appendix S1: exd70153‐sup‐0001‐AppendixS1.docx. Table S1: Baseline characteristics of biomarker population as part of the Phase2b IASOS trial. Table S2: List of proteins excluded from statistical analysis of OLINK data. Proteins with over 90% of measurements below limit of detection were excluded from the analysis. Table S3: Outcomes of statistical tests conducted for full cohort. Data for heatmap in Figure 2A. BL, Baseline; FCH, foldchange; LS, Lesional; NL, Non‐lesional; SEM, standard error of mean; W16, Week 16. Table S4: Outcomes of statistical tests stratified by PASI75 response. Data for heatmap in Figure S3. BL, Baseline; FCH, foldchange; LS, Lesional; NL, Non‐lesional; SEM, standard error of mean; W16, Week 16. Table S5: Outcomes of statistical tests stratified by PASI90 response. Data for Figure 3B. FCH, foldchange; SE, standard error. Table S6: Number of differential expressed proteins in PASI75 Responders and Non‐responders. Differential expressed proteins were defined as |FCH| > 1.2 and false‐discovery rate < 0.05 for OLINK proteins. For IL23, a p value < 0.05 was used as a cutoff criterion, given that IL23 was quantified with a different methodology as it is not part of the OLINK 96 Inflammation panel. Figure S1: Sample flow of tape strips for IL23 analysis. Week 16 samples were only analysed for treatment completers. BL, Baseline; BLOD, below limit of detection; W16, Week 16. Figure S2: Differential expressed proteins before and after treatment with orismilast and placebo. Differential expressed proteins were defined as |FCH| > 1.2 and false‐discovery rate < 0.05 for OLINK proteins. For IL23, a p value < 0.05 was used as a cutoff criterion, given that IL23 was quantified with a different methodology as it is not part of the OLINK Inflammation panel. Figure S3: Heatmap of biomarker response in PASI75 Responders and Non‐Responders. Heatmap is displaying fold changes of proteins which are elevated at baseline. BL, Baseline; NL, Nonlesional; LS, lesional; [file EXD-34-e70153-s001.docx]

**Supplementary Information**

**Orismilast, a potent and selective PDE4B/D inhibitor, reduces protein levels of key disease driving cytokines in the skin of patients with plaque psoriasis**

Richard B. Warren,^1^ Anne Weiss,^2^ Jakob Felding,^2^ Morten O.A. Sommer,^2,3^ Sandra Garcet,^4^ James G. Krueger^4^

^1^ Dermatology Centre, Salford Royal NHS Foundation Trust, Manchester NIHR Biomedical Research Centre, The University of Manchester, Manchester, UK

^2^ UNION Therapeutics A/S, Hellerup, Denmark

^3^ Novo Nordisk Foundation Center for Biosustainability, Technical University of Denmark (DTU), Lyngby, Denmark

^4^ Laboratory of Investigative Dermatology, The Rockefeller University, New York, New York, USA

**Correspondence**

James G. Krueger, Laboratory of Investigative Dermatology, The Rockefeller University, 1230 York Ave # 178, New York, NY 10065, USA. Email: [kruegej@rockefeller.edu](mailto:kruegej@rockefeller.edu)

**Supplementary Table 1 : Baseline characteristics of biomarker population as part of the Phase2b IASOS trial.**

|  | **Placebo** | **20mg bid** | **30mg bid** |
| --- | --- | --- | --- |
| **Biomarker Population** | | | |
| **ITT population with baseline lesional tape strip sample** | 44 | 40 | 48 |
| PASI**,** mean (median) | 19.5 (17.7) | 19.6 (19.0) | 19.9 (16.6) |
| **Treatment completers with Week 16 tape strip sample** | 28 | 28 | 30 |
| PASI**,** mean (median) | 20.4 (17.9) | 21.3 (19.8) | 21.9 (19.5) |
| **ITT Population^1^** | | | |
| Number | 51 | 48 | 50 |
| PASI, median | 17.4 | 19.3 | 16.9 |

^1^ as published in Warren RB, et al., Orismilast in moderate-to-severe psoriasis: Efficacy and safety from a 16-week, randomized, double-blinded, placebo-controlled, dose-finding, and phase 2b trial (IASOS). J Am Acad Dermatol. 2023:S0190-9622(23)03187-0.

**Supplementary Table 2 : List of proteins excluded from statistical analysis of OLINK data**. Proteins with over 90% of measurements below limit of detection were excluded from the analysis.

| **Protein** |
| --- |
| GDNF |
| IL.2RB |
| IL2 |
| SLAMF1 |
| FGF.23 |
| FGF.5 |
| FGF.21 |
| Beta.NGF |
| IL.24 |
| IL13 |
| IL33 |
| IL4 |
| NRTN |
| CCL25 |
| IL5 |
| IL7 |
| CCL11 |
| IL.10RA |
| LIF.R |
| ARTN |
| IL10 |
| NT.3 |

**Supplementary Table 3: Outcomes of statistical tests conducted for full cohort.**Data for heatmap in Figure 2A. BL = Baseline; FCH = foldchange, NL = Non-lesional, LS = Lesional, W16 = Week 16, SEM = standard error of mean

| **Protein** | **Placebo** | | | | **Orismilast 20 mg** | | | | **Orismilast 30 mg** | | | |
| --- | --- | --- | --- | --- | --- | --- | --- | --- | --- | --- | --- | --- |
|  | BL.LS vs. BL.NL | | W16.LS vs. BL.LS | | BL.LS vs. BL.NL | | W16.LS vs. BL.LS | | BL.LS vs. BL.NL | | W16.LS vs. BL.LS | |
|  | FCH | SEM | FCH | SEM | FCH | SEM | FCH | SEM | FCH | SEM | FCH | SEM |
| IL8 | 1.62 | 0.22 | -1.25 | 0.26 | 1.17 | 0.23 | -1.07 | 0.26 | 1.14 | 0.22 | -1.20 | 0.25 |
| VEGFA | 1.59 | 0.19 | -1.31 | 0.23 | 2.07 | 0.20 | -1.43 | 0.23 | 1.33 | 0.19 | -1.23 | 0.22 |
| CD8A | -2.67 | 0.17 | 1.50 | 0.21 | -2.12 | 0.18 | 1.42 | 0.21 | -2.12 | 0.17 | 1.56 | 0.20 |
| MCP3 | 1.06 | 0.12 | 1.03 | 0.15 | 1.44 | 0.13 | -1.19 | 0.15 | 1.22 | 0.12 | -1.16 | 0.14 |
| CDCP1 | 1.54 | 0.16 | -1.10 | 0.19 | 1.61 | 0.17 | 1.06 | 0.19 | 1.52 | 0.16 | -1.35 | 0.18 |
| CD244 | -1.11 | 0.09 | 1.02 | 0.11 | 1.07 | 0.10 | 1.04 | 0.11 | -1.01 | 0.09 | 1.00 | 0.11 |
| OPG | -1.52 | 0.16 | -1.04 | 0.19 | -1.27 | 0.17 | 1.01 | 0.19 | -1.13 | 0.16 | 1.18 | 0.18 |
| TGFbeta | -1.10 | 0.13 | 1.19 | 0.15 | -1.01 | 0.13 | 1.01 | 0.15 | -1.22 | 0.13 | 1.23 | 0.15 |
| uPA | -1.35 | 0.14 | -1.00 | 0.16 | -1.12 | 0.14 | 1.15 | 0.16 | -1.00 | 0.13 | 1.25 | 0.16 |
| IL6 | 1.48 | 0.16 | -1.19 | 0.19 | 1.62 | 0.17 | -1.17 | 0.19 | 1.51 | 0.16 | -1.03 | 0.18 |
| IL17C | 4.08 | 0.24 | -1.50 | 0.28 | 3.37 | 0.25 | -1.93 | 0.29 | 3.05 | 0.24 | -2.37 | 0.27 |
| MCP1 | -3.34 | 0.23 | 1.37 | 0.27 | -2.41 | 0.24 | 1.50 | 0.28 | -1.84 | 0.23 | 1.62 | 0.26 |
| IL17A | 2.74 | 0.21 | -1.21 | 0.25 | 1.95 | 0.22 | -1.51 | 0.26 | 1.99 | 0.21 | -1.50 | 0.24 |
| CXCL11 | 1.14 | 0.13 | -1.02 | 0.15 | 1.32 | 0.13 | -1.33 | 0.15 | 1.27 | 0.13 | -1.18 | 0.15 |
| AXIN1 | -1.83 | 0.14 | 1.26 | 0.17 | -1.51 | 0.15 | 1.15 | 0.17 | -1.65 | 0.14 | 1.24 | 0.16 |
| TRAIL | -1.62 | 0.13 | -1.04 | 0.15 | -1.63 | 0.14 | 1.15 | 0.16 | -1.41 | 0.13 | 1.17 | 0.15 |
| IL20RA | -2.00 | 0.16 | 1.48 | 0.19 | -2.01 | 0.17 | 1.27 | 0.19 | -1.83 | 0.15 | 1.47 | 0.18 |
| CXCL9 | 3.45 | 0.22 | -1.34 | 0.26 | 3.09 | 0.23 | -1.51 | 0.27 | 2.68 | 0.22 | -1.99 | 0.25 |
| CST5 | -10.73 | 0.38 | 1.61 | 0.45 | -12.00 | 0.40 | 2.28 | 0.46 | -14.83 | 0.38 | 5.24 | 0.43 |
| IL1alpha | -12.74 | 0.33 | 1.93 | 0.39 | -18.45 | 0.35 | 3.07 | 0.39 | -15.45 | 0.32 | 4.93 | 0.37 |
| OSM | 4.52 | 0.33 | -1.81 | 0.38 | 2.61 | 0.34 | -1.55 | 0.39 | 4.08 | 0.32 | -1.81 | 0.37 |
| CXCL1 | 2.52 | 0.23 | -1.33 | 0.27 | 1.50 | 0.24 | -1.27 | 0.28 | 1.35 | 0.23 | -1.24 | 0.26 |
| TSLP | 1.38 | 0.12 | -1.31 | 0.14 | 1.43 | 0.12 | -1.41 | 0.14 | 1.69 | 0.12 | -1.56 | 0.13 |
| CCL4 | 1.64 | 0.19 | -1.13 | 0.22 | 1.26 | 0.20 | -1.54 | 0.23 | 1.39 | 0.19 | -1.44 | 0.21 |
| CD6 | 1.22 | 0.08 | 1.08 | 0.09 | 1.34 | 0.08 | -1.02 | 0.09 | 1.26 | 0.08 | -1.14 | 0.09 |
| SCF | -1.64 | 0.10 | 1.24 | 0.12 | -1.52 | 0.11 | 1.11 | 0.12 | -1.39 | 0.10 | 1.42 | 0.11 |
| IL18 | 3.54 | 0.27 | 1.00 | 0.32 | 2.83 | 0.28 | -1.46 | 0.32 | 3.59 | 0.26 | -2.06 | 0.30 |
| TGFa | 1.10 | 0.15 | -1.22 | 0.18 | 1.39 | 0.16 | -1.29 | 0.18 | 1.33 | 0.15 | -1.55 | 0.17 |
| MCP4 | -3.22 | 0.19 | 1.05 | 0.22 | -1.73 | 0.20 | 1.16 | 0.23 | -1.75 | 0.19 | -1.04 | 0.21 |
| TNFSF14 | 1.08 | 0.18 | 1.02 | 0.21 | 1.16 | 0.19 | 1.08 | 0.22 | 1.28 | 0.18 | -1.01 | 0.20 |
| MMP1 | 3.20 | 0.29 | -1.77 | 0.35 | 3.78 | 0.31 | -1.59 | 0.35 | 4.84 | 0.29 | -1.75 | 0.33 |
| CCL19 | -1.15 | 0.20 | -1.08 | 0.23 | -1.12 | 0.21 | 1.05 | 0.24 | -1.13 | 0.20 | 1.37 | 0.23 |
| IL15RA | -4.70 | 0.25 | 2.02 | 0.29 | -4.21 | 0.26 | 1.75 | 0.30 | -5.13 | 0.25 | 2.80 | 0.28 |
| IL10RB | -1.67 | 0.11 | 1.21 | 0.14 | -1.31 | 0.12 | 1.01 | 0.14 | -1.40 | 0.11 | 1.12 | 0.13 |
| IL22RA1 | -1.07 | 0.21 | 1.03 | 0.25 | -1.03 | 0.22 | 1.34 | 0.25 | -1.30 | 0.20 | 1.41 | 0.24 |
| IL18R1 | -1.42 | 0.12 | 1.13 | 0.14 | -1.22 | 0.12 | 1.21 | 0.14 | -1.33 | 0.12 | 1.15 | 0.13 |
| PDL1 | 2.43 | 0.16 | -1.29 | 0.19 | 2.15 | 0.17 | -1.20 | 0.19 | 1.86 | 0.16 | -1.31 | 0.18 |
| CXCL5 | -1.47 | 0.19 | -1.28 | 0.23 | -1.41 | 0.20 | 1.03 | 0.23 | -1.39 | 0.19 | 1.14 | 0.22 |
| TRANCE | -1.28 | 0.13 | 1.25 | 0.15 | -1.17 | 0.13 | 1.26 | 0.15 | -1.00 | 0.13 | 1.05 | 0.14 |
| HGF | 1.17 | 0.16 | -1.13 | 0.19 | 1.07 | 0.17 | 1.14 | 0.19 | 1.39 | 0.16 | 1.04 | 0.18 |
| IL12B | 4.68 | 0.24 | -1.60 | 0.29 | 2.96 | 0.26 | -1.32 | 0.29 | 2.92 | 0.24 | -2.12 | 0.28 |
| MMP10 | -1.26 | 0.24 | -1.17 | 0.28 | -1.41 | 0.25 | 1.21 | 0.29 | -1.31 | 0.23 | 1.48 | 0.27 |
| TNF | 1.54 | 0.15 | -1.16 | 0.17 | 1.56 | 0.16 | -1.32 | 0.18 | 1.45 | 0.15 | -1.29 | 0.17 |
| CCL23 | 1.38 | 0.11 | -1.01 | 0.13 | 1.35 | 0.11 | -1.08 | 0.13 | 1.21 | 0.11 | -1.15 | 0.12 |
| CD5 | 1.09 | 0.14 | 1.16 | 0.17 | 1.31 | 0.15 | 1.10 | 0.17 | 1.10 | 0.14 | 1.02 | 0.16 |
| CCL3 | 1.92 | 0.18 | -1.18 | 0.21 | 1.57 | 0.19 | -1.84 | 0.22 | 1.61 | 0.18 | -2.07 | 0.21 |
| Flt3L | -1.49 | 0.18 | 1.22 | 0.21 | -1.13 | 0.19 | 1.40 | 0.22 | -1.16 | 0.18 | 1.67 | 0.21 |
| CXCL6 | 2.27 | 0.21 | -1.45 | 0.25 | 1.76 | 0.23 | -1.53 | 0.26 | 2.51 | 0.21 | -1.79 | 0.24 |
| CXCL10 | 4.24 | 0.27 | -1.30 | 0.32 | 3.51 | 0.28 | -1.54 | 0.32 | 3.17 | 0.26 | -2.01 | 0.30 |
| X4EBP1 | 6.74 | 0.28 | -1.46 | 0.33 | 4.59 | 0.29 | -2.13 | 0.33 | 3.68 | 0.27 | -2.88 | 0.32 |
| IL20 | -1.94 | 0.14 | 1.18 | 0.17 | -1.73 | 0.15 | 1.22 | 0.17 | -1.77 | 0.14 | 1.33 | 0.16 |
| SIRT2 | 4.45 | 0.22 | -1.34 | 0.26 | 4.34 | 0.24 | -1.78 | 0.27 | 3.32 | 0.22 | -2.32 | 0.25 |
| CCL28 | -1.25 | 0.10 | 1.03 | 0.11 | -1.21 | 0.10 | 1.03 | 0.12 | -1.04 | 0.10 | 1.03 | 0.11 |
| DNER | -12.72 | 0.29 | 2.00 | 0.35 | -12.85 | 0.31 | 2.75 | 0.35 | -10.72 | 0.29 | 3.47 | 0.33 |
| ENRAGE | -1.33 | 0.19 | 1.06 | 0.22 | -2.51 | 0.20 | 1.46 | 0.23 | -2.17 | 0.19 | 1.67 | 0.21 |
| CD40 | 1.08 | 0.17 | 1.20 | 0.20 | 1.50 | 0.18 | -1.04 | 0.20 | 1.07 | 0.17 | -1.35 | 0.19 |
| IFNg | 1.11 | 0.20 | 1.13 | 0.23 | 1.14 | 0.21 | -1.20 | 0.24 | 1.33 | 0.19 | -1.48 | 0.22 |
| FGF19 | -1.14 | 0.09 | -1.01 | 0.10 | -1.06 | 0.09 | -1.02 | 0.10 | -1.14 | 0.08 | 1.27 | 0.10 |
| LIF | -2.90 | 0.16 | 1.24 | 0.19 | -2.42 | 0.17 | 1.37 | 0.20 | -2.38 | 0.16 | 1.49 | 0.19 |
| MCP2 | 1.12 | 0.14 | -1.08 | 0.17 | 1.27 | 0.15 | -1.27 | 0.17 | 1.28 | 0.14 | -1.35 | 0.16 |
| CASP8 | 1.32 | 0.14 | 1.05 | 0.17 | 1.26 | 0.15 | -1.10 | 0.17 | 1.14 | 0.14 | -1.08 | 0.16 |
| CX3CL1 | -3.96 | 0.24 | 1.10 | 0.28 | -3.30 | 0.25 | 1.72 | 0.29 | -2.62 | 0.24 | 1.85 | 0.27 |
| TNFRSF9 | 1.70 | 0.13 | 1.03 | 0.15 | 1.53 | 0.13 | -1.00 | 0.15 | 1.37 | 0.13 | -1.03 | 0.14 |
| TWEAK | -1.53 | 0.15 | 1.04 | 0.18 | -1.79 | 0.16 | 1.41 | 0.18 | -1.17 | 0.15 | 1.29 | 0.17 |
| CCL20 | 4.69 | 0.26 | -1.36 | 0.31 | 3.96 | 0.27 | -1.76 | 0.31 | 3.19 | 0.26 | -2.08 | 0.30 |
| ST1A1 | -3.28 | 0.20 | 1.57 | 0.23 | -3.10 | 0.21 | 1.47 | 0.24 | -3.26 | 0.19 | 1.90 | 0.22 |
| STAMBP | 1.62 | 0.13 | -1.03 | 0.15 | 1.69 | 0.14 | -1.32 | 0.16 | 1.37 | 0.13 | -1.51 | 0.15 |
| ADA | -2.97 | 0.26 | 1.24 | 0.31 | -2.58 | 0.27 | 2.13 | 0.31 | -2.28 | 0.26 | 1.71 | 0.29 |
| TNFB | -1.16 | 0.10 | 1.06 | 0.11 | -1.08 | 0.10 | -1.02 | 0.12 | -1.06 | 0.10 | 1.01 | 0.11 |
| CSF1 | -1.07 | 0.13 | 1.06 | 0.16 | -1.13 | 0.14 | 1.31 | 0.16 | -1.15 | 0.13 | 1.40 | 0.15 |

**Supplementary Table 4: Outcomes of statistical tests stratified by PASI75 response**Data for heatmap in Supplementary Figure 3. BL = Baseline; FCH = foldchange, NL = Non-lesional, LS = Lesional, W16 = Week 16, SEM = standard error of mean

| **Protein** | **PASI75 Non-Responder** | | | | | | | **PASI75 Responder** | | | | | | |
| --- | --- | --- | --- | --- | --- | --- | --- | --- | --- | --- | --- | --- | --- | --- |
|  | BL.LS.vs.  BL.NL | W16.LS.vs.BL.LS | | | | | | BL.LS.vs. BL.NL | W16.LS.vs.BL.LS | | | | | |
|  |  | Placebo | | Orismilast 20 mg | | Orismilast 30 mg | |  | Placebo | | Orismilast 20 mg | | Orismilast 30 mg | |
|  | FCH | FCH | SEM | FCH | SEM | FCH | SEM | FCH | FCH | SEM | FCH | SEM | FCH | SEM |
| VEGFA | 1.64 | -1.31 | 0.28 | -1.40 | 0.32 | 1.41 | 0.38 | 1.45 | -1.24 | 0.43 | -1.52 | 0.33 | -1.67 | 0.26 |
| CDCP1 | 1.51 | -1.12 | 0.20 | 1.07 | 0.23 | -1.20 | 0.27 | 1.48 | -1.16 | 0.44 | 1.01 | 0.34 | -1.46 | 0.26 |
| IL18 | 3.30 | -1.11 | 0.33 | 1.19 | 0.37 | -1.59 | 0.44 | 3.33 | 1.43 | 0.77 | -3.22 | 0.58 | -2.56 | 0.45 |
| CCL23 | 1.32 | 1.00 | 0.15 | 1.09 | 0.17 | -1.04 | 0.20 | 1.35 | 1.06 | 0.28 | -1.37 | 0.21 | -1.21 | 0.17 |
| IL17C | 3.57 | -1.44 | 0.31 | -1.52 | 0.35 | -1.73 | 0.41 | 3.63 | -1.30 | 0.61 | -2.60 | 0.46 | -2.77 | 0.36 |
| IL6 | 1.55 | -1.14 | 0.21 | 1.04 | 0.24 | 1.02 | 0.29 | 1.77 | -1.28 | 0.43 | -1.42 | 0.32 | -1.01 | 0.25 |
| MMP1 | 4.23 | -1.87 | 0.40 | -1.05 | 0.46 | -1.69 | 0.55 | 4.57 | -1.27 | 0.66 | -2.25 | 0.50 | -1.63 | 0.39 |
| CXCL6 | 2.20 | -1.41 | 0.28 | -1.63 | 0.32 | -1.58 | 0.38 | 2.12 | -1.75 | 0.57 | -1.37 | 0.43 | -1.89 | 0.34 |
| CXCL10 | 3.86 | -1.20 | 0.34 | -1.16 | 0.39 | -1.23 | 0.46 | 3.59 | -1.95 | 0.74 | -2.33 | 0.56 | -2.50 | 0.44 |
| CCL3 | 1.78 | -1.11 | 0.24 | -1.67 | 0.27 | -2.00 | 0.32 | 1.64 | -1.85 | 0.48 | -2.02 | 0.36 | -2.19 | 0.28 |
| CXCL9 | 3.11 | -1.21 | 0.29 | -1.08 | 0.34 | -1.45 | 0.40 | 3.07 | -1.86 | 0.60 | -2.55 | 0.45 | -2.34 | 0.35 |
| IFNgamma | 1.18 | 1.23 | 0.27 | -1.11 | 0.31 | -1.32 | 0.36 | 1.01 | -1.22 | 0.49 | -1.31 | 0.37 | -1.57 | 0.29 |
| CCL4 | 1.48 | -1.07 | 0.26 | -1.47 | 0.30 | -1.34 | 0.36 | 1.37 | -1.49 | 0.47 | -1.53 | 0.36 | -1.49 | 0.28 |
| CXCL11 | 1.27 | 1.02 | 0.17 | -1.13 | 0.20 | 1.14 | 0.23 | 1.15 | -1.36 | 0.33 | -1.64 | 0.25 | -1.42 | 0.20 |
| TNF | 1.54 | -1.13 | 0.21 | -1.28 | 0.24 | -1.17 | 0.28 | 1.57 | -1.17 | 0.33 | -1.33 | 0.25 | -1.35 | 0.20 |
| IL12B | 3.42 | -1.51 | 0.30 | 1.04 | 0.34 | -1.58 | 0.40 | 3.66 | -1.94 | 0.65 | -2.11 | 0.49 | -2.55 | 0.38 |
| IL17A | 2.28 | -1.17 | 0.28 | -1.19 | 0.32 | 1.07 | 0.38 | 2.10 | -1.27 | 0.54 | -1.98 | 0.41 | -1.91 | 0.32 |
| CCL20 | 4.05 | -1.28 | 0.34 | -1.22 | 0.39 | -2.07 | 0.46 | 4.04 | -1.55 | 0.67 | -2.75 | 0.50 | -2.26 | 0.39 |
| CXCL1 | 1.64 | -1.22 | 0.32 | -1.44 | 0.37 | -1.70 | 0.43 | 1.71 | -1.82 | 0.57 | -1.10 | 0.43 | -1.11 | 0.34 |
| TSLP | 1.52 | -1.16 | 0.17 | -1.26 | 0.19 | -1.32 | 0.23 | 1.46 | -1.76 | 0.26 | -1.58 | 0.20 | -1.69 | 0.16 |
| TNFRSF9 | 1.59 | 1.07 | 0.16 | 1.00 | 0.19 | -1.26 | 0.22 | 1.42 | -1.06 | 0.36 | -1.07 | 0.27 | 1.08 | 0.21 |
| X4EBP1 | 5.28 | -1.36 | 0.35 | -1.40 | 0.41 | -2.11 | 0.48 | 4.81 | -1.57 | 0.76 | -3.71 | 0.57 | -3.56 | 0.45 |
| SIRT2 | 4.34 | -1.29 | 0.28 | -1.33 | 0.32 | -1.64 | 0.38 | 3.75 | -1.27 | 0.64 | -2.60 | 0.48 | -2.95 | 0.38 |
| OSM | 3.54 | -1.48 | 0.44 | -1.17 | 0.51 | -1.94 | 0.60 | 4.18 | -3.51 | 0.79 | -2.01 | 0.60 | -1.63 | 0.47 |
| MCP2 | 1.20 | -1.18 | 0.19 | -1.18 | 0.21 | -1.12 | 0.25 | 1.20 | 1.22 | 0.37 | -1.31 | 0.28 | -1.43 | 0.22 |
| PDL1 | 2.26 | -1.26 | 0.21 | -1.05 | 0.24 | -1.46 | 0.28 | 2.03 | -1.58 | 0.44 | -1.50 | 0.34 | -1.28 | 0.26 |
| STAMBP | 1.58 | 1.01 | 0.17 | -1.20 | 0.20 | -1.30 | 0.23 | 1.54 | -1.05 | 0.36 | -1.54 | 0.27 | -1.69 | 0.21 |
| TGFalpha | 1.30 | -1.29 | 0.20 | -1.05 | 0.23 | -1.36 | 0.27 | 1.28 | -1.10 | 0.42 | -1.60 | 0.32 | -1.64 | 0.25 |
| CD6 | 1.31 | 1.06 | 0.12 | -1.05 | 0.13 | -1.10 | 0.16 | 1.25 | 1.15 | 0.17 | 1.00 | 0.13 | -1.19 | 0.10 |
| MCP3 | 1.26 | 1.01 | 0.17 | -1.03 | 0.20 | -1.07 | 0.24 | 1.24 | 1.12 | 0.28 | -1.41 | 0.21 | -1.17 | 0.17 |
| CASP8 | 1.30 | 1.10 | 0.19 | 1.04 | 0.22 | -1.15 | 0.26 | 1.27 | -1.01 | 0.37 | -1.32 | 0.28 | -1.03 | 0.22 |
| HGF | 1.19 | -1.13 | 0.22 | -1.01 | 0.25 | -1.12 | 0.30 | 1.20 | -1.21 | 0.42 | 1.43 | 0.32 | 1.18 | 0.25 |

**Supplementary Table 5: Outcomes of statistical tests stratified by PASI90 response.** Data for Figure 3B. FCH = foldchange, SE = standard error

| **Protein** | **PASI90 Non-responder** | | **PASI90 Responder** | |
| --- | --- | --- | --- | --- |
|  | **Log2 FCH** | **SE** | **Log2 FCH** | **SE** |
| CCL20 | -2.29 | 0.50 | -4.43 | 0.77 |
| IL-17A | -2.09 | 0.39 | -2.96 | 0.59 |
| IL-17C | -2.57 | 0.45 | -3.73 | 0.69 |
| IL-18 | -2.12 | 0.49 | -4.51 | 0.75 |
| IL-23 | -1.19 | 0.35 | -2.55 | 0.52 |
| TNFα | -1.91 | 0.32 | -2.91 | 0.49 |
| VEGF-A | -2.07 | 0.42 | -3.62 | 0.64 |

**Supplementary Table 6: Number of differential expressed proteins in PASI75 Responders and Non-responders.** Differential expressed proteins were defined as |FCH| > 1.2 and false-discovery rate < 0.05 for OLINK proteins. For IL23, a p value < 0.05 was used as a cutoff criterion, given that IL23 was quantified with a different methodology as it is not part of the OLINK 96 Inflammation panel.

| **Treatment** | **PASI75**  **Responder** | **PASI75**  **Non-responder** |
| --- | --- | --- |
| Placebo | 0 | 1 |
| Orismilast 20mg bid | 15 | 0 |
| Orismilast 30mg bid | 17 | 0 |


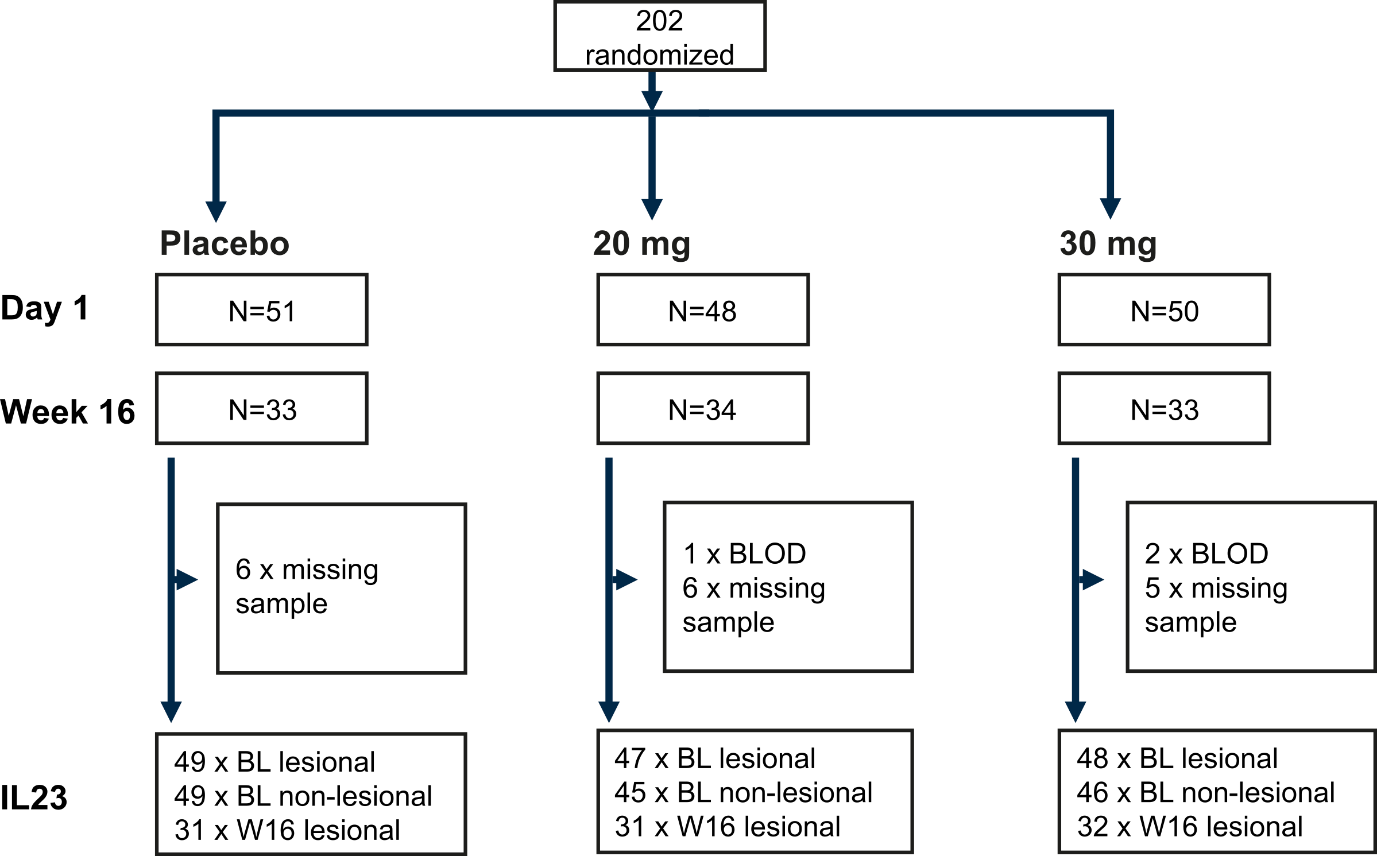


**Supplementary Figure 1: Sample flow of tape strips for IL23 analysis**. Week 16 samples were only analyzed for treatment completers. BL = Baseline, W16 = Week 16. BLOD = below limit of detection

**
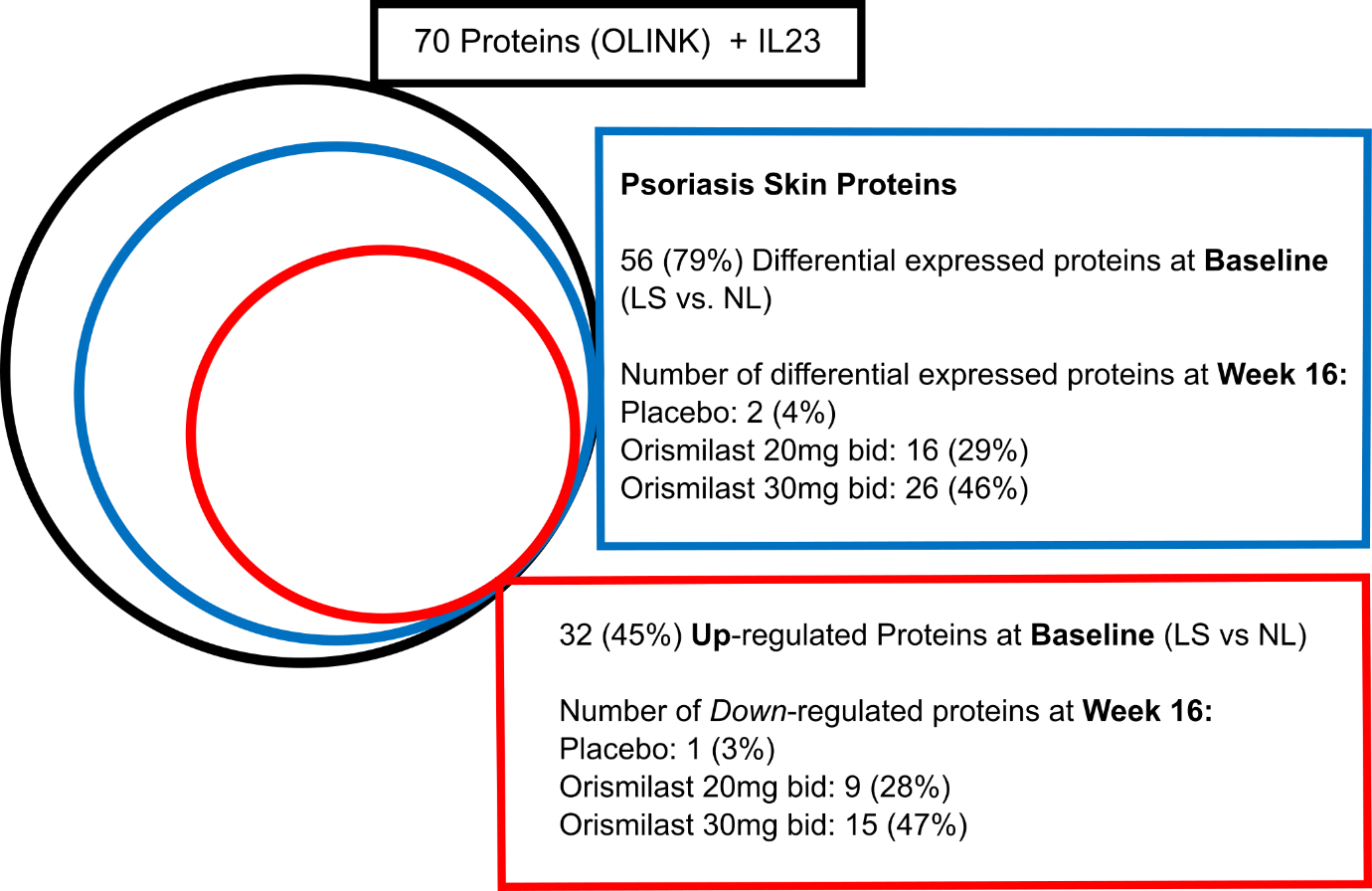
**

**Supplementary Figure 2: Differential expressed proteins before and after treatment with orismilast and placebo**. Differential expressed proteins were defined as |FCH| > 1.2 and false-discovery rate < 0.05 for OLINK proteins. For IL23, a p value < 0.05 was used as a cutoff criterion, given that IL23 was quantified with a different methodology as it is not part of the OLINK Inflammation panel.


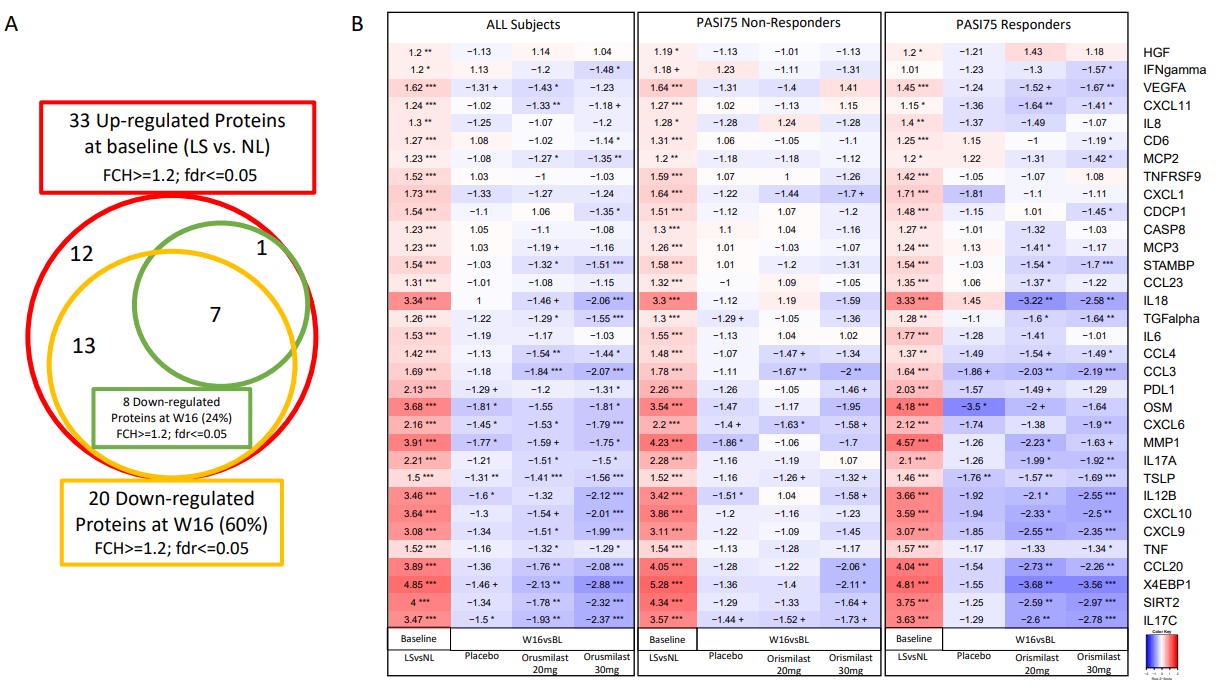


**Supplementary Figure 3:** **Heatmap of biomarker response in PASI75 Responders and Non-Responders**. Heatmap is displaying fold changes of proteins which are elevated at baseline. BL = Baseline, LS = lesional, NL = Nonlesional, W16 = Week 16.
